# Loss Aversion Reflects Information Accumulation, Not Bias: A Drift-Diffusion Model Study

**DOI:** 10.3389/fpsyg.2017.01708

**Published:** 2017-10-10

**Authors:** Summer N. Clay, John A. Clithero, Alison M. Harris, Catherine L. Reed

**Affiliations:** ^1^Department of Behavioral and Organizational Sciences, Claremont Graduate University, Claremont, CA, United States; ^2^Department of Economics, Pomona College, Claremont, CA, United States; ^3^Department of Psychology, Claremont McKenna College, Claremont, CA, United States

**Keywords:** loss aversion, drift-diffusion model, information processing, decision making

## Abstract

Defined as increased sensitivity to losses, loss aversion is often conceptualized as a cognitive bias. However, findings that loss aversion has an attentional or emotional regulation component suggest that it may instead reflect differences in information processing. To distinguish these alternatives, we applied the drift-diffusion model (DDM) to choice and response time (RT) data in a card gambling task with unknown risk distributions. Loss aversion was measured separately for each participant. Dividing the participants into terciles based on loss aversion estimates, we found that the most loss-averse group showed a significantly lower drift rate than the other two groups, indicating overall slower uptake of information. In contrast, neither the starting bias nor the threshold separation (barrier) varied by group, suggesting that decision thresholds are not affected by loss aversion. These results shed new light on the cognitive mechanisms underlying loss aversion, consistent with an account based on information accumulation.

## Introduction

Numerous studies of decision-making have found that losses hurt more than equivalent gains feel good (Kahneman et al., 1990). Kahneman and Tversky (1979) characterized this “loss aversion” as an overweighting of the subjective value of loss outcomes relative to gain outcomes. For example, in risky decision-making, loss aversion typically manifests as risk aversion for mixed gambles that have equally probable outcomes of gain and loss. That is, when people are loss averse, they generally avoid risk by rejecting 50–50 gambles to win or lose, unless the amount to win is twice as much as the amount to lose. This finding has been replicated repeatedly over the last 30 years (Kahneman and Tversky, 1979; Tversky and Kahneman, 1991; Tom et al., 2007; Camerer, 2012).

For the most part, loss aversion has been discussed in descriptive terms: individual decision makers avoid loss, and thus are loss averse. However, few researchers have formally modeled risky decision making to explore the underlying cognitive factors that contribute to loss-averse behavior (c.f. Usher and McClelland, 2004). Existing studies can be characterized as conceptualizing loss aversion in one of two ways: either as a *cognitive bias* in avoiding losses (Kahneman et al., 1990), or in terms of differences in *information processing* arising from an attentional or emotional regulation component (Tom et al., 2007; Sokol-Hessner et al., 2009, 2015; de Martino et al., 2010). Here, we use a model of the choice process (i.e., a drift-diffusion model or DDM) to formally assess which of these cognitive mechanisms better account for loss aversion in risky decision making.

Cognitive bias in the decision process may be conceptualized as an internal decision strategy that biases the individual toward a particular response. Using the terminology of signal detection theory (Green and Swets, 1974; Macmillan and Creelman, 1991), this would correspond to a response bias or criterion shift, changing the likelihood that a decision-maker selects one option over the other. For example, in a two-alternative forced choice (2AFC) task, an observer is required to take one of two options in response to a stimulus on every trial (e.g., “Accept” or “Reject” in response to a gamble). In the absence of any bias, we would expect the two alternatives to be chosen with equal frequency. A cognitive bias would shift the response criterion, making the observer favor one of the two responses. In this context, loss aversion can be thought of as a cognitive bias against changes that make things worse than they currently are: e.g., “a reluctance to accept a loss on any dimension” (Kahneman et al., 1990, p. 1345). In signal detection terms, this would shift the criterion so that the observer becomes more likely to choose one of the responses (i.e., the alternative that makes a loss outcome less probable) over the other. Individuals with high loss aversion would have conservative criteria for avoiding loss and thus, have a strong tendency to reject choices with loss. On the other hand, individuals low on loss aversion would have liberal criteria for avoiding loss and display less of a tendency to reject choices with loss.

If cognitive bias fully explains loss aversion, then loss-averse behavior would always occur in decision contexts with losses. Yet loss-averse behavior is absent in many decision contexts involving losses (Mellers et al., 1999; Erev et al., 2008; Yechiam and Hochman, 2013b, 2014). For example, decisions do not generate behavior consistent with loss aversion when they involve money already intended to be given up for the purchase of a good (Novemsky and Kahneman, 2005), repetitive losses and gains (Erev et al., 2008), or multiple unit-holdings (Burson et al., 2013). Thus, the mere existence of a threat of loss does not necessarily mean an individual will display loss-averse behavior.

Instead, research suggests that loss aversion arises from differences in processing of information about losses while the decision is being made. Recent studies have indicated that emotional responses during decision making may influence the extent to which an individual displays loss-averse behavior in decision contexts with loss (Sokol-Hessner et al., 2009, 2013, 2015), and that interoceptive ability, or the ability to perceive one's own emotions, correlates with one's degree of loss aversion (Sokol-Hessner et al., 2015). Other research examining information search suggests that exploration behavior is increased in the presence of losses, relative to gains (Lejarraga et al., 2012; Lejarraga and Hertwig, 2017), which the authors attribute to increased vigilance or attention when the threat of loss is present. Collectively, these findings suggest that *how decision-relevant information is evaluated in loss contexts* may play a role in loss-averse behavior.

To investigate the extent to which loss aversion depends on cognitive bias vs. information processing, here we employed a computational modeling approach in conjunction with a novel gambling task. This paradigm, based on the Columbia Card Task (CCT; Figner et al., 2009), is characterized by consequential repeated decisions in which decision-makers do not know the underlying risk distribution and must learn from the outcomes of past choices (Barron and Erev, 2003), consistent with real-world decision making. Additionally, this task provides a dynamic decision environment, in which choices vary with respect to risk (possible outcome magnitude) and outcome probability. We combined the choice and response time (RT) data from this task with a DDM (Ratcliff, 1978; Ratcliff and McKoon, 2008), a prominent model of the choice process. In addition to having been successfully used to fit data from other economic tasks (e.g., Krajbich et al., 2010; Milosavljevic et al., 2010; Philiastides and Ratcliff, 2013), the DDM is particularly relevant to our question because it has separable parameters mapping onto cognitive bias and information processing. The DDM posits that, to make a decision, choice values are compared and evaluated over time until a threshold is reached for a particular choice—a process often called evidence accumulation. In the model, evidence refers to any information that is used to compare and evaluate alternative choices. The DDM assumes that evidence accumulation is an inherently noisy process, an idea supported by neural evidence (Cavanagh et al., 2011; van Maanen et al., 2011; Mulder et al., 2012).

Specifically, the DDM uses choice and RT data to separate out a set of parameters corresponding to different cognitive processes: *starting point bias* for which one response is initially favored over another; *drift rate* which indexes the strength or quality of stimulus information; and threshold separation (*barrier*) which is linked to response caution and speed/accuracy trade-offs (Ratcliff, 1978; Ratcliff and Rouder, 2000; Ratcliff and Tuerlinckx, 2002; Ratcliff and McKoon, 2008). The starting point parameter reflects *a priori* biases present at the start of the decision process that favor a particular choice over the other. The drift rate parameter captures the average rate of evidence accumulation toward one choice over the other, thus reflecting the strength of information favoring one option over another. The stronger the relative evidence toward one option, the faster the average RT. Therefore, more difficult tasks have smaller drift rate values (i.e., slower rates, less steep slopes toward one choice over the other) and easier tasks have larger drift rate values. In the context of value-based choice, such as, whether to choose a risky gamble or not, the weighting and integration of choice attributes (e.g., probability or magnitude of outcome) into a relative preference measure is assumed to be captured by the drift rate (Krajbich et al., 2010; Milosavljevic et al., 2010). The barrier parameter reflects the amount of information needed to reach a decision. In a two-choice task, the barrier is the distance between the two alternative choice thresholds. Generally, the barrier parameter reflects instructed trade-offs in emphasizing speed vs. accuracy, or maximizing a reward rate for a given duration of time. In addition to these cognitive parameters, the DDM includes a non-decision time parameter to reflect perceptual and mnemonic processes preceding choice, i.e., non-decision relevant processing (Ratcliff et al., 2016).

Modeling behavioral choice and RT data using the DDM allows us to parse out whether observed choices consistent with loss aversion can be attributed to bias, information processing, or response caution in risky decision making. More importantly, the DDM allows us to make several predictions about which cognitive factors in risky decision making are affected by loss aversion. If loss aversion reflects an individual's bias in avoiding losses, we would expect the starting point parameter to vary with respect to the degree of loss aversion. For example, individuals with high loss aversion may have a greater tendency to take action to minimize bad outcomes relative to individuals without high loss aversion. However, if loss aversion is driven by informational processing differences rather than a cognitive bias, we would expect these differences to separate by drift rate parameter. Specifically, if evaluative and attentional processes contribute to a reduction in loss-averse behavior, then loss-averse individuals may have greater difficulty engaging in those evaluative and attentional processes in a decision context where losses can occur. In the DDM, greater difficulty in the information processing of losses would be reflected by smaller drift rates for individuals scoring high on loss aversion compared to the drift rates of individuals scoring low on loss aversion. Finally, loss aversion may affect the barrier parameter. If loss aversion is a bias in ensuring accuracy of responses, then loss aversion could affect the speed of motor responses. For example, loss aversion may paralyze or slow a decision-maker from making any decision when there is a possibility of a loss. Loss aversion is typically reduced under time pressure (Kocher et al., 2013; Saquib and Chan, 2015), supporting the idea that the degree of loss aversion may separate the barrier parameter.

Therefore, in this experiment we applied a DDM in order to distinguish between these competing cognitive explanations for loss aversion. Participants were classified as being low, moderate, or high in loss aversion based on their responses to an independent assessment instrument for the measurement of risk propensity, the Dynamically Optimized Sequential Experiments (DOSE; Wang et al., unpublished). Following the DOSE, participants completed a computerized gambling task, the modified CCT. To characterize basic behavioral differences across participants, we ran a hierarchical linear regression analysis on choice and RT data, identifying significant variation in behavior between groups. Finally, the DDM was used to relate these group-level behavioral differences to computational parameters associated with specific cognitive processes and computations, thereby enriching our fundamental understanding of this behavioral phenomenon.

## Methods

### Participants

A total of 107 healthy young adults between the ages of 18–22 (*M* = 19.56, *SD* = 1.44; 45 female) were recruited from Claremont McKenna College psychology classes. Participants were given course credit in exchange for volunteering and the opportunity to be placed in a lottery for a gift card based on task performance. This study was reviewed and approved by the Claremont McKenna College Institutional Review Board. All participants provided informed and written consent prior to the experiment and were debriefed following the experiment. Three participants were removed from analysis based on the number of missed responses (more than 10% of trials). Thus, 104 participants were entered into the reported analyses.

### Procedure

First, participants completed the DOSE instrument as a measure of risk propensity (Wang et al., unpublished). The DOSE is a computerized bank of lottery questions from Holt and Laury (2002) and Sokol-Hessner et al. (2009) questionnaires. For each lottery question, participants chose either a sure amount of loss or gain or a probability-based amount of loss or gain (e.g., a 50% chance of winning $4 or losing $2 vs. a 100% probability of gaining $1). Using the participant's responses to past questions, the DOSE employs a Bayesian procedure to select future questions to quickly calculate the utility function expressing the participant's subjective value of gains and losses, risk aversion (rho), loss aversion (lambda), and choice consistency as done in Sokol-Hessner et al. (2009). Each participant's utility functions are calculated separately for gains and losses, with parameters derived for loss aversion, risk aversion, and choice consistency. Loss aversion is indexed by the “lambda” parameter **(Appendix)**. If lambda is equal to one, then gains and losses are equally valued. The use of the DOSE with its Bayesian question-selection procedure requires only about 40 questions to assess loss aversion, risk aversion, and choice consistency and can be completed within 5–10 min; in contrast to what would normally be a 110-item paper-and-pencil questionnaire taking approximately 30–40 min to complete.

**Figure 1:**
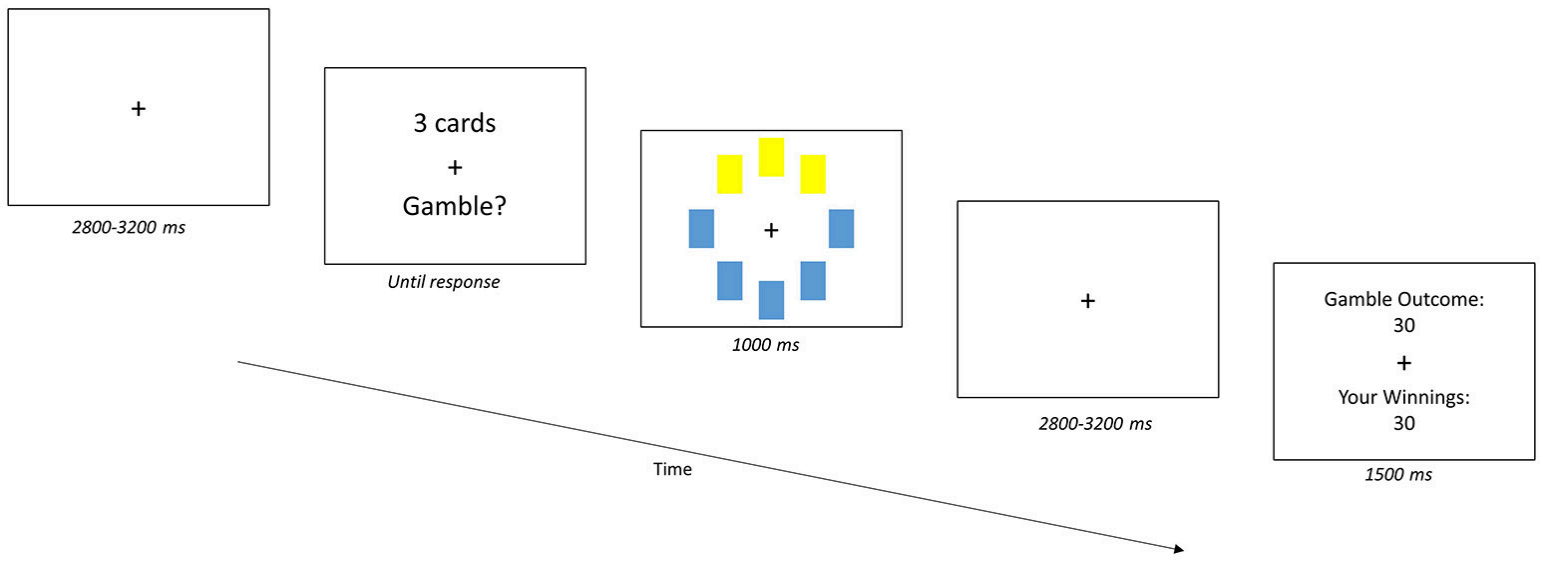
Gambling Task. Participants accepted or rejected a gamble of cards from a 32-card deck with card values of either +10 or −10. The gambles (trials) began with the presentation of a jittered (2,800–3,200 ms) fixation cross. Next, the number of cards to be turned over for the gamble (“X cards”) was presented. For each gamble, eight cards from the deck are randomly selected, and then one, three or five cards could be turned over and the participant indicated their choice of accepting or rejecting the gamble. Participants could accept or reject the gamble using one of four options to indicate the confidence in their response: Strong Yes, Weak Yes, Weak No, and Strong No. The response was followed by a visual of the gamble and then a second jittered fixation cross. Participants then received feedback on the outcome of the gamble and their winnings, following their response and presentation of the gamble.

Next, all participants completed a computerized gambling task (Figure 1) that was modified from the CCT (Figner et al., 2009). The modified CCT is different from the original CCT in that the number of cards for each gamble is fixed at one, three, or five cards, selected by the computer, and participants choose whether they want to accept or reject the gamble. These modifications allow us to systematically manipulate the degree of risk and map the paradigm onto a 2AFC task. Participants received the following instructions: (1) they would be presented with a deck of cards and some number of cards would be selected from the deck for each trial or “gamble”; (2) each card had a value of either +10 or −10; and (3) their task was to decide to accept or reject the gamble. The amount that they would win or lose on that trial would be sum of the values on the selected cards (e.g., for a 3-card gamble with two positive cards (+20) and one negative card (−10), the gamble outcome would be +10).

To provide both “win” and “loss” environments, participants participated in two separate gambling runs, each with a different virtual 32-card deck: the “win” deck had a 55% probability of a positive-value card and the “loss” deck had a 45% probability of a positive-value card. Participants were never told the outcome probabilities for either deck. When playing with each deck, participants received eight cards randomly selected from the deck on each trial. Risk was manipulated by the number of cards turned over for each gamble (gamble type): one (least risky), three, or five (most risky). Each gamble type occurred with equal frequency.

Gamble trials began with a jittered (2,800–3,200 ms) fixation cross, followed by a screen indicating the number of cards to be turned over for the gamble (e.g., “5 cards”). With the card values unknown, participants either “accept” or “reject” the gamble; they indicated their confidence in their “accept” or “reject” response by pressing one of four keys: Strong Yes, Weak Yes, Weak No, and Strong No. After giving their response, participants were shown which cards were turned over and, following a jittered fixation cross (2,800–3,200 ms), participants received feedback on the gamble outcome regardless of their response. Participants only won or lost points based on the outcome *if they accepted the gamble*. After each trial, the cards were put back into the deck for the next gamble. At the end of the experiment, participants were asked to select the deck they would prefer if they were randomly chosen to play one more block of gambles. Participants were awarded with a monetary amount that equaled a weighted sum of their earnings across both decks.

For both the win and the loss deck, each participant completed three blocks of 24 gambles, with eight trials of each gamble type in each block. Thus, each deck had 72 gambles and there was a total of 144 trials. Deck-type order was counterbalanced across participants.

### Analysis procedures

RTs were positively skewed, but modeling with a logarithmic transform of RT did not substantively change the results. Therefore, untransformed values of RTs were used as the dependent variable for better interpretability. Trials with RTs <200 ms were excluded from all analysis, as outlier RT can be problematic for DDM fitting (Ratcliff and Tuerlinckx, 2002). A hierarchical, or multilevel regression model, was fit to RT data in place of a standard repeated-measures analysis of variance (ANOVA). A drift diffusion model was fit to RT and choice data to parcel out latent cognitive parameters underlying the time course of the decision process.

#### Hierarchical model

RT data was submitted to a hierarchical regression model to determine any within-subject RT differences in the gambling task. The analysis was implemented using the nlme package in R (Pinheiro et al., 2016). Unlike standard repeated-measures ANOVAs, this analysis avoids violating assumptions of homogeneity of regression, independence of errors, or sphericity (Quené and Van Den Bergh, 2004). The base model was built to start with the most theoretically important predictor and subsequent predictors were added in order of importance (Raudenbush and Bryk, 2002). Predictors that did not enhance prediction (i.e., no difference in model fit with addition of the predictor) were dropped, unless they were components of cross-level interactions.

#### Drift-diffusion model

To investigate the connection between loss aversion and decision time, a hierarchical DDM was estimated. The hierarchical Bayesian model estimates parameters for each individual participant, but those individual estimates are governed by group-level means and variances. DDM estimation was performed using a freely available software package in Python (Wiecki et al., 2013). The models were estimated using a Bayesian hierarchical framework, with Markov chain Monte-Carlo (MCMC) sampling methods employed to estimate a joint posterior distribution of the model parameters. An important benefit of hierarchical Bayesian estimation is that it outperforms other methods for fitting DDM specifications, especially when there are a fairly limited number of trials (Wiecki et al., 2013). Estimation used non-informative priors: there were no a priori assumptions and all priors were uniform distributions over large intervals of possible parameter values. Additional details on the likelihood function used to estimate the DDM are provided in Wiecki et al. (2013). Using Gibbs sampling, a common MCMC algorithm, 11,000 samples were drawn from the posterior, with the first 1,000 discarded as burn-in.

**Figure 2:**
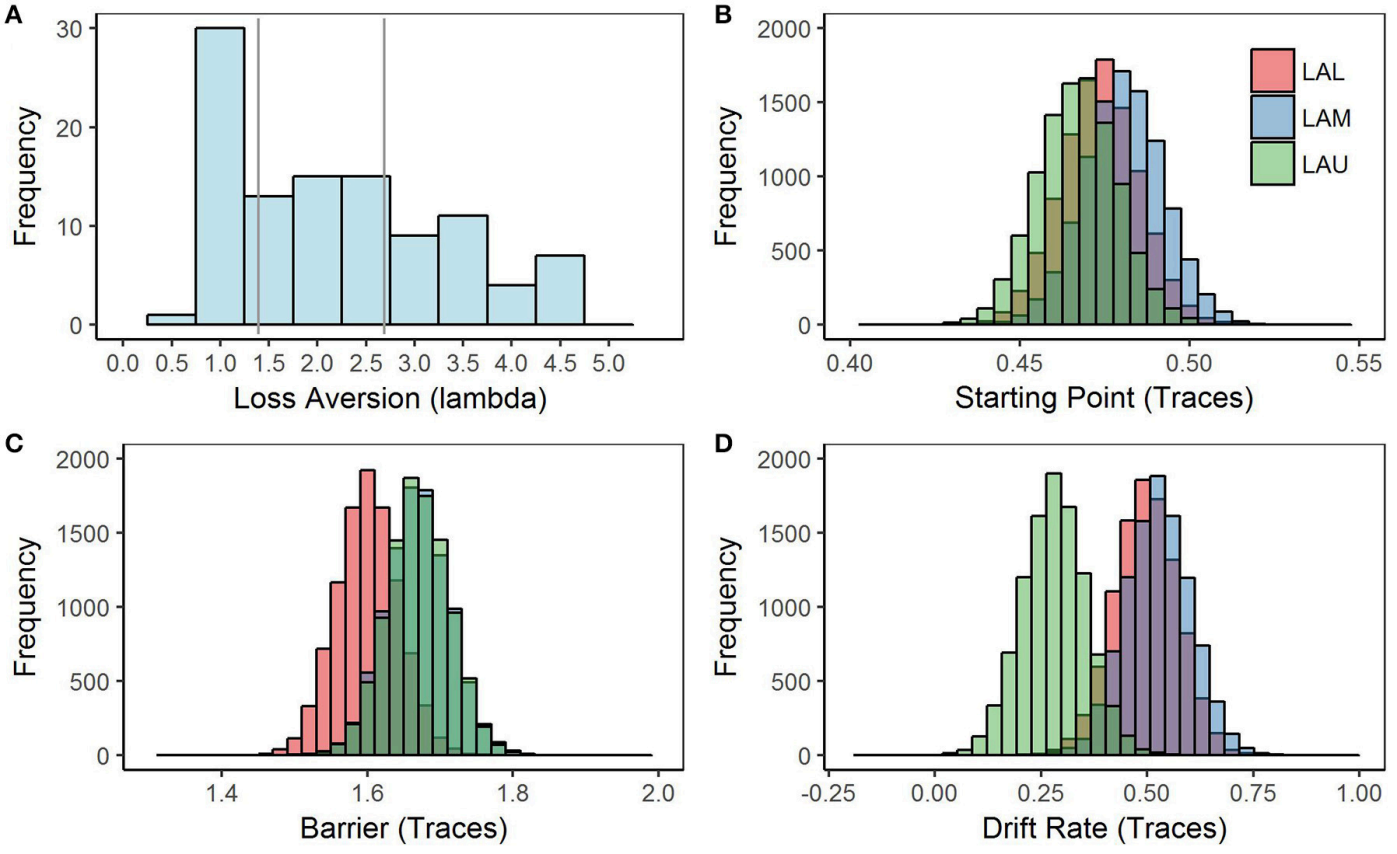
LA Histogram and Model 1 posterior distributions for each parameter by loss aversion group. Model 1 was estimated using a Bayesian hierarchical framework, with Markov chain Monte-Carlo (MCMC) sampling methods employed to estimate a joint posterior distribution (known as *traces*) for each of the model parameters, starting point, barrier, and drift rate. **(A)** Histogram of loss aversion scores with gray lines separating each tercile group. Our participant sample was more loss averse (*M* = 2.17, *SD* = 1.14), as measured by the DOSE using the lambda (λ) parameter than samples in the literature; although, our study had the benefit of a larger sample size and fewer exclusions than is typical. Generally, scores on the DOSE where lambda > 1 are considered loss-averse and scores below 1 are considered loss-seeking. **(B)** Posterior distribution of starting point traces estimated by Model 1. Starting point traces are separated by the loss aversion groups. **(C)** Posterior distribution of barrier traces estimated by Model 1. Although barrier traces seem to somewhat separate based on loss aversion groups, the difference between groups is not significant. **(D)** Posterior distribution of drift rate traces estimated by Model 1. Drift rate traces are separated by the loss aversion groups, *p* < 0.05.

Individual differences in loss aversion were implemented as a group variable in the model. Participants were binned into three groups based on tercile loss aversion scores as measured by the DOSE: upper, middle, and low loss aversion. The upper loss aversion group (LAU) included participants who had lambda scores higher than 2.69 (group average = 3.53). The middle loss aversion group (LAM) included participants who had lambda scores between 1.39 and 2.69 (group average = 2.07). The low loss aversion group (LAL) included participants who had lambda scores lower than 1.39 (group average = 0.979). Approximately 25% of our entire participant population have scores below 1.0 which would classify them as loss-seeking. The low tercile group (LAL) included these “loss-seeking” individuals as well as those individuals with lambdas close to 1.0 (see Figure 2A). The distribution of our sample was right skewed for loss aversion, as is typical of loss aversion scores. Our sample was slightly more loss-averse than others reported in the literature (*M* = 2.17, *SD* = 1.13), but this difference may be accounted for by the large sample size in our study.

All participants were fit simultaneously in a single hierarchical DDM, but the model assumed three different group-level distributions of parameters, based on the loss-aversion groups. Specifically, the hierarchical model fit a separate drift rate (v), starting point (z), threshold (a), and non-decision time parameter for each subject. The choice related parameters (drift rate, starting point, and threshold) were all allowed to vary by loss aversion group. Bayesian hypothesis testing was then performed on the estimated group posterior means to determine if there are any significant differences across loss aversion groups. Model fit and model comparison were assessed using the Deviance Information Criterion, or DIC (Spiegelhalter et al., 2014).

## Results

### Overall results

Overall, participants demonstrated a bias to accept rather than reject gambles (*Percent Accept* = 63.1 vs. *Percent Reject* = 36.9). A one-way ANOVA on RTs with the within-subjects factor block (1, 2, 3) showed that mean RTs decreased over blocks [*F*_(2, 106)_ = 20.45, *p* < 0.001, η_*p*_^2^ = 0.16]. Although we did not assess the learning of the underlying risk distribution directly, participants were able to infer the properties of the two decks because when asked with which deck they would select if they were randomly selected to play one more block, all participants chose the win deck. This suggests that they could infer which deck had more winning cards despite the close win/loss ratios between the decks (45/55 vs. 55/45).

### Hierarchical model

To investigate how risk and proportion of wining cards affected RTs for choices in the gambling task, we conducted a two-level hierarchical model using maximum likelihood estimation to assess the effects of choice type (accept, reject), confidence (confident, non-confident), deck type (win, loss), and gamble type (1-, 3-, 5-cards) on RT. First-level units were single trials in the gambling task, with 14,687 trial observations included in the analysis. Second-level units were the 104 participants. In the final reported model, trials and individual participants were declared random-effects to assess both the variability among trials within individuals and the variability among individuals. All other variables were included as fixed-effect predictors. The intraclass correlation of 0.22 indicated that approximately 22% of the variance in RTs was due to differences between individual participants; thus, modeling participants as a random effect was appropriate^1^. The final model that provided the best fit of the data (−*2logLik* = 210475, *AIC* = 210503, *BIC* = 210609) is reported (Table 1).

**Table 1:** Summary of hierarchical model 1.

**Fixed Effects**	**Null model**		**Random slopes model**		**Final model**	
	**β**	***SE***	**β**	***SE***	**β**	***SE***
Intercept	878.50	16.90	931.00	16.85	925.40	16.98
Time (Trial)			−1.48	0.22	−1.46	0.21
Accept					−24.02^**^	7.11
Confidence					−116.08^***^	11.46
Deck					18.79^**^	5.09
Card 3					−12.77	7.94
Card 5					42.68^***^	8.89
Accept × Confidence					−3.57	11.10
Confidence × Card 3					−9.72	13.39
Confidence × Card 5					−54.28^***^	12.91
**Random Effects**	**Estimate**	***SE***	**Estimate**	***SE***	**Estimate**	***SE***
Residual error	101,575	318	98,987	315	94,727	308
Intercepts	28,995	170	26,779	164	27,170	165
Slopes			3.32	1.82	3.06	1.75

^*^p < 0.05;

** p < 0.01;

*** *p < 0.001*.

Participants were generally faster for “accept” responses compared to “reject” responses and for confident choices compared to non-confident choices. RTs for accepting gambles were 13 ms faster on average than RTs for rejecting gambles, β_*Accept*_ = −24.02, *SE* = 7.11, 95% CI = [−10.09, −37.96], *t*_(14, 574)_ = −3.38, *p* < 0.001. Similarly, RTs for confident choices were 116 ms faster than RTs for non-confident choices, β_*Confident*_ = −116.08, *SE* = 11.46, 95% CI = [−138.54, −93.61], *t*_(14, 574)_ = −10.12, *p* < 0.0001. There was no interaction effect between choice type (accept vs. reject) and confidence on RTs, β_A*C_ = −3.57, *SE* = 11.10, 95% CI = [−25.31, 18.17], *t*_(14, 574)_ = −0.32, *p* = 0.75, indicating that confidence did not affect RTs for accepting or rejecting gambles. RTs for the win deck were 18 ms slower than RTs for the loss deck, β_*Win*_ = 18.79, *SE* = 5.09, 95% CI = [8.82, 28.76], *t*_(14, 574)_ = 3.69, *p* < 0.001.

Risky gambles produced slower overall RT, with RTs for 5-card gambles 42 ms slower than RTs for 1-card gambles, β_5*Card*_ = 42.68, *SE* = 8.89, 95% CI = [25.25, 60.11], *t*_(14, 574)_ = 4.80, *p* < 0.0001. However, this only held for non-confident choices. Confident choices for the most risky, 5-card gambles were actually faster than those for the least risky, 1-card gambles; RTs of confident choices for 5-card gambles were 54 ms *faster* on average than non-confident choices for 1-card gambles, β_5*C*∗*C*_ = −54.28, *SE* = 12.91, 95% CI = [−79.58, −28.98], *t*_(14, 574)_ = −4.20, *p* < 0.0001. RTs for 3-card gambles were not significantly faster than RTs for 1-card gambles and there was no interaction with confidence, β_3*Card*_ = −12.77, *SE* = 7.94, 95% CI = [−28.33, 2.79], *t*_(14, 574)_ = −1.61, *p* = 0.11; β_3*C*∗*C*_ = −9.72, *SE* = 13.39, 95% CI = [−35.96, 16.52], *t*_(14, 574)_ = −0.73, *p* = 0.47.

To investigate how loss aversion might influence RTs, a second hierarchical model was conducted on RT data with the three loss aversion groups as a fixed effect predictor: upper (LAU), middle (LAM), and low (LAL). The HLM specifications and procedure for the second model were the same as for the first model. For all gamble types, participants in the LAU group were slower to respond, by approximately 100 ms on average, compared to participants in the LAL group, β_*Int*_ = 880.98, *SE* = 27.68, 95% CI = [826.72, 935.23]; β_*LAU*_ = 99.72, *SE* = 38.88, 95% CI = [175.92, 23.52], *t*_(101)_ = 2.56, *p* < 0.05. No significant differences were found between the LAM and LAL groups, β_*LAM*_ = 54.90, *SE* = 39.15, 95% CI = [131.65, −21.83], *t*_(101)_ = 1.40, *p* = 0.16.

In summary, participants had an overall bias to accept gambles and RTs for accepted gambles were faster than those for rejected gambles. Additionally, confidence interacted with risky decision times: RTs were slowest for the riskiest gambles when confidence was low, but fastest for these gambles when confidence was high. Finally, we found significant differences in the speed of response for the highly loss-averse group, compared to the moderate or low loss aversion groups, indicating that participants' degree of loss aversion plays a role in the decision process in the task.

Although these results provide initial support for differences in decision behavior linked to loss aversion, the hierarchical model approach is blind to the nature of the mental operations driving these behavioral differences between groups. Therefore, we further evaluated choice and RT data together in terms of group loss aversion differences with a hierarchical drift diffusion model to map choice and RT differences onto specific underlying cognitive factors.

### DDM

To investigate the cognitive mechanisms underlying loss aversion, we fit a DDM to the joint distribution of choice and RT data for participants, using the three loss-aversion groups to dictate group-level parameters. We estimated four separate parameters: the starting point, drift rate, barrier, and the non-decision time. The starting point parameter indicates bias toward one choice over the other at the start of a trial. Therefore, if loss aversion is driven by a cognitive bias, starting point may vary with respect to the loss aversion groups. If loss aversion affects the weighing and processing of information, then the drift rate parameter may reflect group differences in loss aversion. Finally, if loss aversion reflects differences in the amount of information needed to initiate a response for one choice over the other, then different loss aversion groups may exhibit differences in the barrier parameter.

#### DDM specification comparisons

We ran three different models to determine the most parsimonious model that best fit the data: (1) a null model; (2) a simple model with parameters split by loss aversion groups only; and (3) a model with the drift rate parameters split by both loss aversion groups and deck type.

The deviance information criterion (DIC) is an appropriate measure for assessing model fit and model comparison for hierarchical Bayesian models (see Wiecki et al., 2013). Across the three models, the lowest DIC was found for model 3, indicating the best fit when drift rate was split by both loss aversion and deck type (*DIC*_1_ = 27,163.5, *DIC*_2_ = 26,579.6, *DIC*_3_ = 26,529.3). An additional means for assessing the model fit is verification that the estimated model can generate the data found in the experiment. To do this, we implemented a posterior predictive check: 100 random draws are taken from the posteriors of the model parameters. The mean squared error (*MSE*) was then computed using the summary statistics for the true data and the summary statistics for each simulated dataset. Specifically, the difference between the observed summary statistic (e.g., percentage of Yes responses) and the simulated summary statistic (e.g., percentage of Yes responses) was squared. The mean across all 100 simulations was the MSE. In all cases, the *MSE* for both choice and RT reflected good fits to the data, and all summary statistics for the simulated data were within 95 percent credible intervals around the observed data. Thus, all models adequately recovered the data. The values are very similar between choice RTs to accept and to reject gambles.

To confirm that the model fits were stable, we assessed model convergence using the $\hat{R}$ statistic (Gelman and Rubin, 1992). A value of $\hat{R}$ = 1 is associated with perfect correspondence across difference chains of the same model, reflecting parameter convergence. For both models, using five different runs of the models, values of $\hat{R}$ were entirely consistent with model convergence (Model 2, average = 1.0001, minimum = 0.9999, maximum = 1.0012; Model 3 = average = 1.0001, minimum = 0.9999, maximum = 1.0010).

#### DDM results

We first examined the effects of loss aversion using a simple model with parameters split by loss aversion group (Table 2). Based on the DIC, this model fit the data significantly better than a model without loss aversion group as a between-subjects factor. We found while neither the starting point (Figure 2B) nor barrier (Figure 2C) varied significantly by degree of loss aversion groups, the drift rate parameter did (Figure 2D). Calculated using the difference in the respective group posteriors, the drift rate was positive and smaller in magnitude for the LAU group relative to the LAM and the LAL groups, *p* < 0.01 and *p* < 0.05. Thus, as smaller drift rates reflect slower time to reach a decision for a given threshold, the upper loss aversion group showed increased processing time to reach a choice relative to the middle and lower loss aversion groups (LAU = 0.28; LAM = 0.53; LAL = 0.50). There were no other differences between the posterior estimates of the starting point and barrier parameters by loss aversion groups, *p* > 0.05. These data support the idea that loss aversion reflects differences in information processing rather than cognitive bias or response caution.

**Table 2:** Simple DDM: parameter means and standard deviations of the trace by loss aversion groups.

	**Drift rate (*μ*)**		**Barrier (*a*)**		**Starting point (*z*)**	
	**AVE**	**SDT**	**AVE**	**SDT**	**AVE**	**SDT**
LAU	0.28	0.07	1.67	0.04	0.47	0.12
LAM	0.53	0.08	1.67	0.04	0.48	0.12
LAL	0.50	0.07	1.60	0.04	0.47	0.11

**Figure 3:**
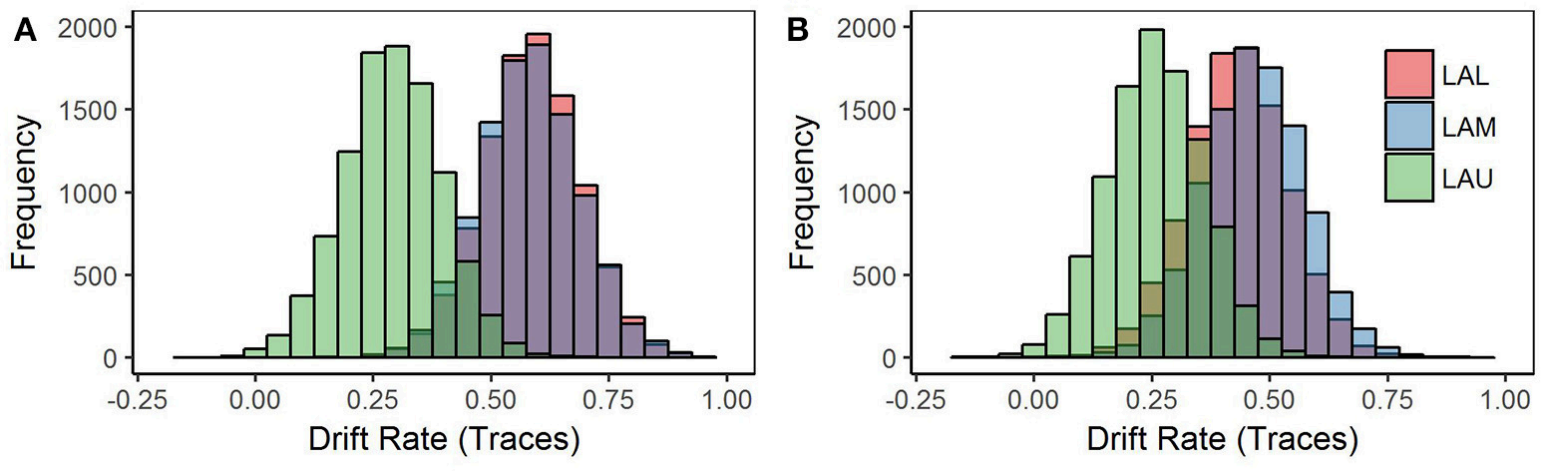
Model 2 posterior distributions for the drift rate parameter by loss aversion groups and deck type. Model 2 was estimated using a Bayesian hierarchical framework, with Markov chain Monte-Carlo (MCMC) sampling methods employed to estimate a joint posterior distribution (known as *traces*) for the model parameters, starting point and barrier, in addition to the drift rate parameter for each deck type, win and loss. The joint posterior distributions of the drift rate parameter for each deck type are displayed here. **(A)** Win deck: Drift rate separates by loss aversion group. **(B)** Loss deck: Drift rate is not separable by loss aversion groups.

To test how loss aversion interacted with the underlying probability distribution of the gambles, we fit a model with the drift rate parameter split by both loss aversion group and deck type (Figure 3). Parsing drift rate by deck type revealed that the differences in drift rate between loss aversion groups are primarily associated with the gain deck, not the loss deck (Table 3). The difference in drift rate between the LAU group and the two groups, LAM and LAL, held for the gain deck at *p* < 0.05 for both tests. However, these differences were not significant for the loss deck. Thus, variance in information processing associated with degree of loss aversion does not reflect a significant difference in attribute weighting in situations where loss is more likely. Instead, the processing of information by highly loss-averse individuals appears to be distinct from less loss-averse individuals *only* in the gain deck, where winning is more likely.

**Table 3:** Full DDM: parameter means and standard deviations of the trace for drift rate by loss aversion groups and deck type.

	**Drift rate (*μ*)**	
	**AVE**	**SDT**
**GAIN DECK**		
LAU	0.29	0.10
LAM	0.58	0.11
LAL	0.59	0.10
**LOSS DECK**		
LAU	0.26	0.10
LAM	0.47	0.11
LAL	0.43	0.10

## Discussion

Although loss aversion has been robustly observed over the last 30 years, the cognitive mechanisms underlying this phenomenon remain largely unexplored. In this study, we used a novel modeling approach to investigate how differences in loss aversion map onto psychological processes, including evidence accumulation, decision bias, and response caution. Because the DDM draws on the full information available from the distribution of RT data rather than simply looking at measures of central tendency, this method can provide superior insight into how different cognitive parameters contribute to response (Voss et al., 2013). Group-level hierarchical linear modeling revealed RT differences associated with loss aversion, namely that highly loss-averse individuals responded more slowly when making gambling decisions compared to less highly loss-averse individuals, on average. Nonetheless, these analyses do not differentiate among the underlying cognitive processes leading to those group differences in RTs. In contrast, the DDM approach revealed that differences in the degree of loss aversion displayed during a risky decision-making task were not reflected in the starting point or barrier parameters of the DDM, but rather in the *drift rate parameter*.

The drift rate represents the rate of an individual's evidence accumulation toward a particular choice, as determined from the perceptual discriminability of the stimulus (Palmer et al., 2005; Ratcliff et al., 2009) or the informational content of the decision (Ratcliff, 1981; Diederich and Busemeyer, 2006). Typically, choices that are perceptually more similar result in drift rates closer to zero, indicating longer time needed to accumulate evidence toward a choice due to weaker stimulus strength (Palmer et al., 2005; van Maanen et al., 2012). Likewise, in choices between different monetary values, the drift rate decreases as the magnitude of difference in payoff values between the two choices decreases (Ratcliff, 1981; Diederich and Busemeyer, 2006).

In our study, we found that the most loss-averse individuals showed the greatest difficulty in choosing between accepting and rejecting gambles, as measured by a smaller drift rate parameter. Furthermore, specifying deck type as a factor in the model revealed that this effect varied with the underlying probability distribution of the gamble: when wins were more likely, the least loss-averse individuals accumulated information at a faster rate. That is, individuals in the low and medium loss-aversion groups accumulated a stronger quality of evidence for the choice to accept gambles in the win deck, relative to both their own performance in the loss deck and to individuals in the highly loss-averse group. These findings suggest that people with higher levels of loss aversion may process risky decision scenarios more slowly and inflexibly, contradicting the idea that loss aversion is driven by pre-existing cognitive or motor response bias.

What could be the mechanism for this greater inflexibility of evidence accumulation in highly loss-averse individuals? Drift rate is known to be affected by attentional salience: that is, time spent looking at a choice is related to greater evidence accumulation for that choice (Krajbich et al., 2010; Krajbich and Rangel, 2011; Cavanagh et al., 2014). The greater the amount of time that individuals fixate on the non-selected option, the greater the difficulty in discriminating between choices, reflected in a smaller drift rate (Krajbich et al., 2010; Krajbich and Rangel, 2011; Cavanagh et al., 2014). Here, we found that separation in the drift rate parameter by loss aversion was only observed for choice scenarios associated with a greater probability of winning (i.e., the gain deck), but not a greater probability of losing (i.e., the loss deck). That is, when the probability of losing is higher, the choice to accept or reject a gamble is less clear because of the decreased likelihood of a winning outcome, regardless of a person's loss aversion score. However, when the probability of winning is higher, the increased potential for receiving a win outcome increases the likelihood of accepting a gamble for people who score in the low or middle range of loss aversion. In contrast, people who score high on loss aversion appear to face difficulty in discriminating between the two options even when the probability of winning is higher.

Given the faster drift rate toward accepting gambles found in the low and middle loss-averse groups, but not the high loss-averse group, what is the underlying cognitive mechanism? Previous research on attentional biases and reward/loss suggests that this result may reflect either (1) an increased attentional bias toward reward in low and middle loss-averse individuals, or (2) increased attentional bias toward loss in highly loss-averse individuals. With respect to the former, high rewards have been shown to drive attentional salience (Hickey and Theeuwes, 2010; Anderson et al., 2011; Hickey and van Zoest, 2012; Theeuwes and Belopolsky, 2012), and the extent to which an individual develops an attentional bias to reward is related to trait reward-seeking (Hickey and Theeuwes, 2010). Thus, one possibility is that highly loss-averse individuals have a smaller attentional bias to reward than those who score at low or mid-range levels, leading to the separation in the drift rate parameter.

On the other hand, highly loss-averse individuals may have an increased attentional bias toward losses, even when rewards are more probable than losses. Loss and threat stimuli have high attentional priority: for example, distractors associated with threat produce slowing of visual search (Schmidt et al., 2015). Previous work has found that both threat and loss-related stimuli capture attention (Müller et al., 2015; Schmidt et al., 2015). Furthermore, whereas loss-related stimuli typically lead to faster disengagement from cued locations compared to neutral stimuli (Bucker and Theeuwes, 2016), high trait anxiety is related to delayed disengagement of attentional capture by negative stimuli (Fox et al., 2002; Verkuil et al., 2009). This is consistent with findings that the threat of losses increases exploratory behavior and information search likely due to increased vigilance or attention (Lejarraga et al., 2012; Lejarraga and Hertwig, 2017). A phenomenon similar to trait anxiety could explain the failure of highly loss-averse individuals in our sample to adjust their evidence accumulation in the win deck due to the continued presence of a potential loss. Future research should look at whether loss averse individuals do not show typical patterns of attention disengagement to losses or increases in information search behavior.

Yet another body of evidence suggests that losses have a strong effect on attention by leading to better task performance (cf. Yechiam and Hochman, 2013a). For example, research using a dual-task paradigm found that performance in a secondary task improved when the primary task contained potential loss outcomes (Yechiam and Hochman, 2014). Likewise, response inhibition develops faster under conditions of punishment compared to conditions of reinforcement (children and tokens: Costantini and Hoving, 1973; adults and monetary outcomes: Dickinson, 2001; Andreoni et al., 2003; Pietras et al., 2010). Such attentional allocation to losses (or lack thereof) can explain whether loss-averse behavior is present or absent in situations with losses (Yechiam and Hochman, 2013b). More specifically, attention to losses may interact with task performance. Yechiam and Hochman (2013a) propose that losses increase on-task attention, which in turn, enhances reinforcement learning and decreases random responding, and this effect is asymmetric depending on whether losses and gains are presented separately or concurrently. Although our experiment examined variation in loss aversion for separate win and loss decks, potential loss and gain outcomes occurred in both conditions. Therefore, future research should address this point by modeling loss aversion in the context of tasks more optimally designed to tease apart reinforcement learning and task demands.

In addition, the use of an unknown risk distribution in our task may have influenced our finding that loss aversion affects the rate of information accumulation rather than starting bias. Because the risk distribution in our experiment was unknown to participants, our experimental set-up may have prioritized information processing over the cognitive processes indexed by other DDM parameters. Future studies looking at the effects of loss aversion on choice processes should manipulate several factors including what information is provided to participants about the risk distribution. Modeling the data from these experiments using a DDM approach could further specify the extent to which different types of information influence the various DDM parameters.

Finally, although we treat loss aversion as a trait in this study, previous research has shown that engaging in emotional regulation can reduce loss aversion within individuals (Sokol-Hessner et al., 2009, 2013, 2015). When participants are instructed to regulate their emotional arousal via a cognitive reappraisal strategy, they show decreases in loss-averse behavior and concomitant reductions in physiological arousal and amygdala activation to losses (Sokol-Hessner et al., 2009, 2013). More recently, loss aversion has been found to correlate with interoceptive ability, or the ability to reliably and accurately perceive one's own emotional reactivity (Sokol-Hessner et al., 2015). Considering these findings, it is an open question whether and how emotional regulation strategies like cognitive reappraisal might modulate the differences in drift rate observed in our most highly loss-averse participants. Given that our sample was more loss-averse than typically reported in the literature, our most loss-averse tercile may have been less able to regulate their emotional arousal or less aware of their own emotional reactivity to loss information in the gain domain. However, there is high individual variability in the ability to regulate emotional responses to loss aversion (Sokol-Hessner et al., 2009). Thus, it may be that trait-level loss aversion and emotional regulation are orthogonal to one another, or both may depend on an additional factor such as, interoception (Sokol-Hessner et al., 2015). Clearly, this question should be addressed in future research.

In summary, the DDM provides a unique approach to formally defining loss aversion in terms of underlying cognitive factors during risky decision-making. Consistent with individual differences in information processing, as opposed to a cognitive bias, these results shed new light on the cognitive mechanisms underlying individual differences in loss aversion. By moving away from a descriptive characterization of loss aversion toward a computational model, our findings provide a foundation for future work to explain and apply loss aversion in terms of information processing within a noisy decision system.

## Author contributions

SC, AH, and CR designed the experiment. SC collected the data. SC and JC analyzed the data. SC, JC, AH, and CR wrote the paper.

### Conflict of interest statement

The authors declare that the research was conducted in the absence of any commercial or financial relationships that could be construed as a potential conflict of interest.

## Footnotes

^1^When trials were included as a random-level predictor nested in individual participants, the intraclass correlation dropped close to zero (*ICC* = −0.09,) indicating that differences between individual participants may be attributable to differences across trials; thus, nesting trials within individuals was appropriate.

## Appendix: estimation of λ in DOSE

According to Wang et al. (unpublished), the DOSE algorithm for estimating loss aversion parameter λ uses the following equation to specify the prospect-theory utility function over losses:

$$\begin{array}{l} u\left(\omega^{-}\right)=-\lambda {\left(-\omega \right)}^{\rho } \end{array}$$

where ω represents the payoff, ρ (risk aversion parameter) characterizes the curvature of the utility function, and λ is the loss aversion parameter. If λ = 1, then gains and losses are valued equally. If λ > 1, then the subject is loss averse and if λ < 1, then the subject is loss seeking. This model is combined with a Bayesian updating procedure to select the most informative set of questions based on participants' previous responses.
